# The Strategic Meaning of CBCA Criteria From the Perspective of Deceivers

**DOI:** 10.3389/fpsyg.2018.00855

**Published:** 2018-06-08

**Authors:** Benjamin G. Maier, Susanna Niehaus, Sina Wachholz, Renate Volbert

**Affiliations:** ^1^Psychologische Hochschule Berlin, Berlin, Germany; ^2^Lucerne University of Applied Sciences and Arts, Lucerne, Switzerland; ^3^Charité - Universitaetsmedizin Berlin, Institute of Forensic Psychiatry, Berlin, Germany

**Keywords:** criteria-based content analysis, strategic self-presentation, content-related deception strategies, beliefs about verbal cues of deception, primary vs. secondary deception, cognitive vs. motivational component

## Abstract

In 2014, Volbert and Steller introduced a revised model of Criteria-Based Content Analysis (CBCA) that grouped a modified set of content criteria in closer reference to their assumed latent processes, resulting in three dimensions of *memory-related, script-deviant* and *strategy-based criteria*. In this model, it is assumed that deceivers try to integrate memory-related criteria—but will not be as good as truth tellers in achieving this—whereas out of strategic considerations they will avoid the expression of the other criteria. The aim of the current study was to test this assumption. A vignette was presented via an online-questionnaire to inquire how participants (*n* = 135) rate the strategic value of CBCA criteria on a five-point scale. One-sample *t*-tests showed that participants attribute positive strategic value to most memory-related criteria and negative value to the remaining criteria, except for the criteria *self-deprecation* and *pardoning the perpetrator*. Overall, our results corroborated the model's suitability in distinguishing different groups of criteria—some which liars are inclined to integrate and others which liars intend to avoid—and in this way provide useful hints for forensic practitioners in appraising the criteria' diagnostic value.

## Introduction

### The empirical footing of CBCA

The underlying rationale of Criteria-Based Content Analysis (CBCA) holds that the content of experienced-based accounts is qualitatively higher than the content of fabricated statements (the so-called Undeutsch Hypothesis; Undeutsch, 1967). After identifying content characteristics that practitioners and scholars deemed suitable to substantiate truthfulness, Steller and Köhnken (1989) compiled a systematic set of 19 CBCA criteria (see Table 1). Since then, a multitude of both field and laboratory studies have confirmed that experience-based accounts indeed yield higher content quality than fabricated statements, in this way corroborating the overall validity of CBCA as a *truth*-detection tool (for recent meta-analyses see Amado et al., 2016; Oberlader et al., 2016). Most of these studies summed up the individual criteria scores to one comprehensive (total) CBCA score, which subsequently served as the relevant variable for further analysis (e.g., Akehurst et al., 2001; Welle et al., 2016). Such an approach, however, may be too simplistic and may underestimate the actual utility of CBCA, since it ignores the possibility that some criteria might be more sensitive to truthfulness and hence bear higher diagnostic value than others. For instance, after having identified children who were able to achieve a higher total CBCA score in their fabricated than in their truthful accounts, Hommers (1997) showed that the criteria *accurately reported details not comprehended, unexpected complications*, and *related external associations* still discriminated between true and fabricated statements. This suggests that particular weights should be assigned to these criteria as predictors of the veracity of statements. More empirical knowledge is essential however, if one intends to advance the prospect of weighting beyond the stage of mere suggestion. To date, CBCA still lacks a weighting system, despite the fact that numerous researchers have criticized its absence and stressed the need for implementation to increase the method's accuracy and sensitivity (e.g., Vrij, 2005; Porter and ten Brinke, 2010).

**Table 1:** Original compilation of CBCA criteria (Steller and Köhnken, 1989).

General characteristics
1. Logical consistency
2. Unstructured Production
3. Quantity of details
Specific contents
4. Contextual embedding
5. Description of interactions
6. Reproduction of conversation
7. Unexpected complication during the incident
Peculiarities of content
8. Unusual details
9. Superfluous details
10. Accurately reported details not comprehended
11. Related external associations
12. Accounts of subjective mental state
13. Attribution of perpetrator's mental state
Motivation-related content
14. Spontaneous corrections
15. Admitting lack of memory
16. Raising doubts about one's own testimony
17. Self-deprecation
18. Pardoning the perpetrator
Offense-specific elements
19. Details characteristic of the offense

### Theoretical considerations about the diagnostic value of CBCA criteria

The diagnostic value of a criterion refers to its validity in discriminating between self-experienced and fabricated statements. For identifying a criterion as diagnostically valuable, its occurrence in true statements is necessary, but by no means sufficient (i.e., Greuel et al., 1998): The relevant question is not how likely a criterion is to occur in true statements, but how likely it is to appear in true *relative* to fabricated statements. As a first step toward inferring the diagnostic value of a criterion, theoretical considerations of what processes govern its emergence loom necessary: Why exactly is a criterion expected to occur in true accounts, but not in fabricated ones? From a psychological perspective, two universal aspects apply to a truth teller but not to a lying person (Volbert and Steller, 2014): Truth tellers report from actual memory, and in doing so are convinced that the event in question had happened as reported. Therefore, in the statement of an honestly reporting person content criteria are likely to occur naturally, as they reflect phenomena associated with genuine memories and feelings of sincerity. A lying person on the other hand needs to put (more) deliberate effort in inventing information that substitutes the missing memory of the alleged experience (creative demands related to *primary deception*; Köhnken, 1990) and in masking the discrepancy between statement and belief by presenting the fabricated event in a credible manner (strategic demands related to *secondary deception*; Köhnken, 1990).

In accordance with the premise of primary vs. secondary deception, Köhnken (1990) distinguished between two forms of CBCA criteria by classifying them as being either cognitively- or motivationally-related. Both kinds of criteria indicate true statements, albeit for different reasons: The former relate to creative (or cognitive) demands; typically, they should be too difficult to produce when fabricating. Motivational criteria refer to how a witness presents a statement; typically, they should be avoided out of strategic considerations when lying. While such categorization suggests that a criterion's diagnostic value is to be derived from either its cognitive or motivational aspects, Niehaus et al. (2005) pointed out that both components need to be taken into account. That is, considerations of the underlying motivational component should also be applied to criteria originally regarded as purely cognitively-related, and vice versa. In summary then, two considerations require clarification if the diagnostic value of a criterion is to be deduced: (1) To what degree is the deceiver inclined to produce the criterion and (2) to what degree would the deceiver be capable of doing so. Insight about the motivational component should hence provide a first hint toward the criterion's diagnostic value: If the deceiver considers the criterion to be strategically detrimental to his or her self-presentation efforts, the likelihood of its emergence in fabricated accounts is generally lower. In turn however, if the deceiver ascribes positive strategic value to a criterion and thus is inclined to produce it, a higher likelihood for its occurrence does not necessarily follow: Whether or not the criterion will emerge should then crucially depend on the cognitive component, that is, how difficult it is for the deceiver to integrate the criterion in his or her statement (*differential controllability*, Köhnken, 1990).

### Previous research about the motivational component

The concept of strategic self-presentation is firmly established within the literature, stating that liars are typically more concerned with appearing credible than truth tellers (DePaulo, 1992; DePaulo et al., 2003). Inquiring about suspects' strategies to appear credible during police interrogations, Hartwig et al. (2007) correspondingly found that lying participants seemed to be more prone to adopting verbal^1^ and non-verbal strategies than truthfully reporting participants. Further scientific efforts to examine deceivers' verbal strategies are hardly existent however. While there are several articles that did elaborate on beliefs of lay people about verbal cues of deception, these studies primarily investigated which kind of contents are believed to be indicative for detecting lies in somebody else's statement (e.g., Granhag and Strömwall, 2000; Vrij et al., 2006; Bogaard et al., 2016). Consequently, insights derived from their findings do not necessarily elucidate on the actual content-related strategies of deceivers, since the results were gained from the perspective of the to-be-deceived rather than from the perspective of the deceiver (Niehaus et al., 2005). That is, while people's beliefs of how lies can be spotted in others are likely to affect their strategies in detecting them (Ryan et al., 2018), these beliefs must not necessarily govern their own way of acting when being deceptive. To our knowledge then, only two studies (Niehaus et al., 2005; Niehaus, 2008) exist which quantitatively examined how laypersons assess the strategic value of CBCA or other content-related criteria in the context of deception. This is certainly surprising, considering that the CBCA criteria classified as motivationally-related are only valid if the forensic assumptions—predicting that laypersons would ascribe negative strategic meaning to these criteria and try to avoid their production when deceiving—are correct. We, therefore, aim to elaborate further on the motivational component of CBCA criteria by building on the findings of Niehaus et al. (2005) and Niehaus (2008) about content-related deception strategies. Because the two previous studies are available in German language only, they are first introduced in more detail.

#### Studies about content-related deception strategies

Both studies asked subjects to take the perspective of a fictitious protagonist, who for personal reasons needed to convincingly lie about a certain type of event. The first investigation (Niehaus et al., 2005; *n* = 120; *M*_age_ = 29.6; all females) presented a scenario in which the protagonist's friend felt unable to press charges against her neighbor, who had previously raped her. Therefore, the protagonist decided to claim that she herself had been raped by the neighbor so that the perpetrator would still receive punishment. The story outline in the second study (Niehaus, 2008; *n* = 50; *M*_age_ = 28.6; 16 men) entailed less severe ramifications: The protagonist needed to find a convincing explanation why he had shown up late at work, as otherwise he would be fired by his boss. In need of an excuse, he wrongly accused his neighbor to have had him trapped in a cellar. After the presentation of the story outline, a standardized questionnaire was handed out on which each CBCA criterion was described in a discrete way as well as illustrated by means of an example embedded into the story outline. Subjects then had to indicate on a 5-point (Niehaus, 2008) or 7-point (Niehaus et al., 2005) scale whether they would rather integrate or avoid the criterion if they aimed to deliver the false statement as convincingly as possible. Negative ratings signified that the criterion was considered to weaken one's credibility, while a positive value indicated that the criterion was believed to promote deception efforts. If a criterion was considered to be strategically irrelevant, the neutral value “0” was to be assigned. For analysis purposes one-sample *t*-tests were conducted to reveal whether the averaged value ratings differed significantly from “0,” indicating that subjects ascribed strategic meaning to the criterion. Table 2 summarizes the obtained results from both studies. Note that some CBCA criteria, as well as their classification, differ from the original compilation (Steller and Köhnken, 1989) since the authors deduced five higher-level strategic goals that deceivers would pursue in the context of sexual rape allegations, and structured the criteria accordingly (for more detail see Niehaus, 2001; Niehaus et al., 2005).

**Table 2:** Strategic value ratings for CBCA criteria^*c*^ (Niehaus et al., 2005; Niehaus, 2008).

**Strategic goal**	**CBCA criterion**	**Classification**
I. Competency (of the deceiver)	**Spontaneous corrections**^a, b^	M (−)
	Admitting lack of memory	M (−)
	**Efforts to remember**^a, b^	M (−)
	**Expressing uncertainty**^a, b^	M (−)
	Reality controls^*a*^	M (−)
	Justifying memory gaps/uncertainties^b^ Spontaneous clarifications	M (+)M (+)
II. Moral impeccability (of the deceiver)	**Raising doubts about one's own person**^a, b^ Self-deprecation^a^	M (−)M (−)
III. Deprecation (of the accused person)	Pardoning the perpetrator^a^	M (−)
IV. Content-related inconspicuousness (of the statement)	Unexpected complications^b^	C (−)
	**Unusual details**^a, b^	C (−)
	Information about everyday-life routines (context)^b^	C (+)
	Spatial information (context)^b^	C (+)
	Temporal information (context)^b^	C (+)
	Description of interactions^a^	C (+)
	Reproduction of conversations^a^	C (+)
	**Emotions and feelings**^a, b^	C (+)
	Attribution of perpetrator's mental state	C (+)
	**Personal implications**^a, b^	C (+)
V. Formal inconspicuousness (of the statement)	**Plausibility**^a, b^	C (+)C (+)C (+)
	**Logical consistency**^a, b^	
	**Quantity of details**^a, b^	
	**Unstructured production**^a, b^	C(−)
	**Superfluous details**^a, b^	C (−)
	Raising doubts about one's own testimony^a^	M (−)

p < 0.05:

a Study of Niehaus et al. (2005)

b *study of Niehaus (2008)*.

*Criteria in green font color received significant positive value ratings in at least one of the two studies, while criteria in red font color symbolize significant negative ratings. Additional bold font was applied if these criteria received significant ratings in both studies*.

*The third row shows whether a criterion was originally classified as cognitively (“C”)- or motivationally-related (“M”), based on the work of Köhnken (1990). Furthermore, Niehaus et al. (2005) referred to the impression-management account to predict in advance whether laypersons would ascribe positive (“+”) or negative (“−”) strategic meaning to a criterion*.

c *To allow meaningful comparisons, criteria that were investigated in only one of the two studies are not presented (self-related/victim-related/neutral associations, attribution of negative traits, clichés, repetitions)*.

As depicted in Table 2, the results give rise to two major implications: First, most motivational criteria were rated as strategically negative and second, cognitive criteria were found to carry strategic meaning as well. The pattern showing that subjects generally ascribed negative strategic meaning to motivational criteria is paramount, given that the discriminatory value of these criteria rests largely on the presumption that laypersons would tend to avoid them in deception contexts. The findings also corroborated the postulation of Niehaus et al. (2005), stating that for each criterion—regardless of its original classification—both motivational and cognitive aspects needed to be considered if its diagnostic value is to be deduced. In regards to the modified structure of the CBCA model (Table 2) however, group I (competency), IV (content-related inconspicuousness) and V (formal inconspicuous) contained criteria of both positive and negative valences. Consequently, the model's structure does not allow for clear group-based distinctions on the motivational level. Considering further that purely motivational aspects governed the classification of the criteria into the five different groups, no information about the cognitive component can be deduced from its criteria groups.

### The revised model of CBCA criteria

In 2014, Volbert and Steller introduced a revised CBCA model, which is based on theoretical considerations of what processes govern the emergence of criteria in statements (Volbert and Steller, 2014). The model still distinguishes criteria pointing to the differences in the cognitive processes of liars and truth tellers (cognitive criteria) from criteria referring to the aspects of strategic self-presentation (motivational criteria). Other than before, cognitive criteria are distinguished even further in the model, resulting in two main groups of cognitively-related criteria: The first group entails details characterizing *episodic autobiographical memory*, such as specific spatiotemporal and self-related information. When deceivers fabricate statements, these characteristics are likely to occur as well since they provide essential information (i.e., *temporal* or *spatial* details) without which any delivered account would appear incomplete. Because liars cannot draw on actual episodic memory however, the criteria are assumed to be expressed in a less elaborate way than in experience-based statements. The second main group of cognitive criteria comprise *script-deviant details*, such as *unusual details* or *unexpected complications during the incident*. Criteria from this group should rarely occur in fabricated accounts, considering that a lying person must construct his or her statement from cognitive scripts (e.g., Schank and Abelson, 1977) to substitute for the lack of experience-based memories. Cognitive scripts reflect the liar's subjective assumptions of what properties typically look like for the event in question (Köhnken, 1990). Script-deviant criteria, on the other hand, refer to characteristics that go beyond the very limitations of such simplified, script-guided knowledge. If the statement giver cannot draw on actual memories providing a potential source for script-deviant elements, he or she should face great difficulties in deliberately producing them as he or she would need to overcome the limited scope of his or her own imagination (Köhnken, 1990). Finally, the third main group refers to motivational criteria, thereby addressing *efforts of positive strategic self-presentation* (see section Theoretical Considerations About the Diagnostic Value of CBCA Criteria for an explanation of why motivational criteria are expected to appear in true rather than fabricated statements). Table 3 depicts the revised model, with the original binary classification of cognitively- vs. motivationally-related criteria presented in brackets behind each criterion.

**Table 3:** Modified system of content characteristics^*a*^ (Volbert and Steller, 2014).

**Autobiographic memory vs. script information**		**Strategic self-presentation**
**Criteria related to episodic autobiographical memory (Group 1)**	**Criteria related to script-deviant information (Group 2)**	**Criteria related to efforts of positive strategic self-presentation (Group 3)**
Information about everyday life routines [C]	Unexpected complications [C]	Spontaneous corrections [M]
Spatial information [C]	Superfluous details [C]	Admitting lack of memory [M]
Temporal information [C]	Unusual details [C]	Efforts to remember [M]
Description of interactions [C]	Related external associations [C]	Expressing uncertainty [M]
Reproduction of conversations [C]	Accurately details not comprehended [C]	Reality controls [M]
Emotions and feelings [C]		Raising doubt about one's own testimony [M]
Own thoughts [C]		Raising doubts about one's own person [M]
Sensory Impressions [C]		Self-deprecation [M]
Attribution of perpetrator's mental state [C]		Pardoning the perpetrator [M]
Personal implications [C]		

*[C], Cognitive criteria; [M], Motivational criteria*.

a *Volbert and Steller (2014) understand their allocation of specific criteria to be exemplary rather than irrevocably. For illustration purposes, the structure presented in this article thus differs slightly from the version originally presented by the authors: In the original version, a separate category “statement as a whole” addresses criteria that can only be evaluated if the statement is analyzed in its entirety, as opposed to scoring the same criterion multiple times at different parts of the statement (“single characteristics”). For reasons of clarity, we exclusively focused on single characteristics and rejected all “statement as a whole” criteria (namely reconstructability of the event, vividness of the event, quantity of details, unstructured production and spontaneous supplementing)*.

### Aim of the present study

The present study inquired about the content-related deception strategies of laypersons like done by Niehaus et al. (2005) and Niehaus (2008) before. However, their categorization of criteria into 5 groups resulted in value ratings that were of opposite valence within some of the groups (i.e., positive *and* negative value ratings across criteria of the same group), thereby rendering group-based distinctions impractical. Against this background, the main goal of the current study was to examine whether the theoretically-driven structure of the revised model would correspond better to the observed pattern of strategic value ratings. Put differently, the relevant question was to what degree the composition of each of the three criteria groups would contain criteria that on the motivational level are compatible with each other (i.e., containing criteria that consistently carry either negative or positive strategic meaning). Derived from the findings of Niehaus et al. (2005) and Niehaus (2008), we expected participants to rate the memory-related (group 1) criteria predominantly positive. In contrast, we expected participants to generally attribute negative strategic value to script-deviant (group 2) and strategy-based (group 3) criteria. Overall, for each of the three criteria groups, the predicted pattern of strategic ratings should result in a degree of compatibility higher than observed for any previous models, meaning that within each group the strategic value ratings should be either consistently negative *or* consistently positive.

## Methods

### Participants

A total of 135 participants (*M*_age_ = 28.6, *SD* = 9.8, Range 19–67; 32 men) filled out a questionnaire^2^ inquiring about content-related deception strategies. The sample consisted mostly of students (*n* = 66) or working professionals (*n* = 55). Participants were recruited via an online participation system of the University of Potsdam (Germany) or through advertisement in public Facebook groups related to psychological topics. Prior to participation, all participants were assured that their data would be treated confidentially, and all participants gave written informed consent. Upon request, credit points were awarded for participation.

### Procedure and material

The survey was administered online by using the platform www.soscisurvey.de. The first items of the questionnaire asked subjects to provide information about age, gender, and occupation. Next, the story outline was presented, with the instruction to assume the perspective of the protagonist. The story closely resembled the one devised by Niehaus (2008), with only minor modifications to preclude potential misunderstandings. In brief, the story^3^ described a protagonist who is at risk of losing his highly valued job, unless he would be able to deliver a convincing explanation to his boss for his (repeatedly) belated arrival at work. After having read the story outline, 27 CBCA criteria derived from the model of Volbert and Steller (2014) were presented on the questionnaire, with the sequence of criteria presentation being adjusted to the model's structure. For each criterion, we gave a short abstract description illustrated by an example embedded into the story outline. For instance, we first described the criterion *spontaneous corrections* by phrasing the question in the following way: “Without being asked, would you refute parts of the information you had already provided at an earlier stage and revise them?” Subsequently, the criterion was illustrated by means of the following story-related example: “Oh no, that was wrong what I had said earlier. In fact, I was holding the folder already in my hands at this time point.” Before the first criterion was presented, we further instructed participants to not pay too much attention to the upcoming examples, but to indicate whether in principle they would rather integrate or avoid the criterion in question. Thereby, we asked participants to also consider to what degree they would feel confident to integrate the criterion in their fabricated accounts. With this instruction, we intended to strengthen participants' motivation to assess the strategic meaning of the criteria in a thorough and attentive way. For each criterion, participants should indicate their strategic assessment on a five-point scale (−2: “No, this would strongly weaken my credibility”; −1: “No, this would weaken my credibility”; 0: “It does not matter. My credibility would remain unchanged”; +1: “Yes, this would strengthen my credibility” +2: “Yes, this would strongly strengthen my credibility”).

## Results

Identical to the investigations of Niehaus et al. (2005) and Niehaus (2008), the goal of our analysis was to compare participants' strategic value ratings for each criterion to the neutral value “0” (= no relevant strategic meaning). Significant differences between any such pair of values would indicate that participants were inclined to either avoid or integrate the respective criterion, dependent on the valence of the criterion's rating (negative vs. positive). We therefore conducted one-sample *t*-tests to assess for each criterion whether its average strategic rating differed significantly at the *p* < 0.05 level from the value “0.” To account for the inflated Type I error rate due to multiple *t*-tests, the Benjamini-Hochberg procedure (Benjamini and Hochberg, 1995) was performed, which controls the expected proportion of falsely rejected null hypotheses (false positives). In consideration of the rather explanatory nature of our study, we preferred this method over Bonferroni corrections, since the latter greatly increase the probability of a Type II error (Narum, 2006). With the false discovery rate set at 5%, no false positives were detected. Consequently, we can safely conclude that at least 95% of the criteria ratings that were found to be significant were correctly identified as such. The results are presented in Table 4, with the criteria structured according to the revised model of Volbert and Steller (2014).

**Table 4:** Mean value, standard deviation, effect size (Cohen's d), 95% confidence interval values and results of the one-sample *t*-test (test value = 0) for each criterion.

	***M***	***SD***	***d***	**95% LCL**	**95% UCL**	t*	***p***
**MEMORY-RELATED CRITERIA [C]**							
Information about everyday-life routines	0.54	0.87	0.62	0.39	0.69	7.22	0.001
Spatial information	0.07	1.01	0.07	−0.11	0.24	0.77	0.222
Temporal information	0.20	1.07	0.19	0.02	0.38	2.17	0.016
Descriptions of interactions	0.01	0.97	0.08	−0.16	0.17	0.9	0.465
Reproduction of conversations	0.00	1.07	0.00	−0.18	0.18	0.00	0.500
Emotions and feelings	0.71	0.98	0.72	0.54	0.88	8.40	0.001
Own thoughts	−0.03	1.08	−0.03	−0.21	0.15	−0.32	0.750^A^
Sensory impressions	−0.16	1.06	−0.15	−0.34	0.03	−1.70	0.092^A^
Attribution of perpetrator's mental state	−0.11	0.90	−0.12	−0.26	0.04	−1.44	0.152^A^
Personal implications	0.46	0.90	0.51	0.31	0.61	5.96	0.001
**SCRIPT-DEVIANT CRITERIA [C]**							
Unexpected complications	−0.34	1.05	−0.33	−0.52	−0.16	−3.79	0.001
Superfluous details	−0.96	0.97	−1.00	−1.13	−0.80	−11.59	0.001
Unusual details	−0.97	1.15	−0.85	−1.17	−0.78	−9.84	0.001
Related external associations	−0.27	1.07	−0.25	−0.45	−0.09	−2.91	0.002
Accurately reported details not comprehended	−0.33	1.06	−0.31	−0.51	−0.15	−3.66	0.001
**STRATEGY-BASED CRITERIA [M]**							
Spontaneous corrections	−0.92	1.09	−0.84	−1.10	−0.73	−9.76	0.001
Admitting lack of memory	−0.21	1.14	−0.19	−0.41	−0.02	−2.20	0.015
Efforts to remember	−0.85	1.08	−0.79	−1.03	−0.67	−9.20	0.001
Expressing uncertainty	−0.41	1.17	−0.35	−0.61	−0.21	−4.06	0.001
Reality controls	−1.02	1.09	−0.94	−1.21	−0.84	−10.91	0.001
Raising doubts about one's own testimony	−0.23	1.20	−0.19	−0.43	−0.03	−2.23	0.014
Raising doubt about one's own person	−1.24	0.96	−1.28	−1.40	−1.07	−14.92	0.001
Self-deprecation	0.36	1.18	0.31	0.16	0.56	3.57	0.001^A^
Pardoning the perpetrator	0.44	1.05	0.42	0.27	0.62	4.93	0.001^A^

* *N = 135; df = 134*.

*LCL, Lower confidence limit; UCL, Upper confidence limit; [C], Cognitive criteria; [M], Motivational criteria*.

*Criteria in green font color received significant positive value ratings, while criteria in red font color symbolize significant negative ratings*.

A *For results contradictory to our hypotheses the p-values from two-tailed tests were reported. Otherwise, the p-values were derived from one-tailed tests, since the testing hypotheses were one directional*.

Viewed irrespective of criteria dimension or classification, the strategic value ratings for 18 of the 24 criteria differed significantly from “0,” indicating that participants considered most criteria to be strategically relevant when deceiving. The effect sizes of the significantly rated criteria ranged from *d* = 0.19 to *d* = 1.28. For 12 of these 18 criteria the value ratings were negative, while the remaining 6 criteria received positive value ratings. In sum then, the criteria can be generally categorized as strategically relevant vs. strategically not relevant from the perspective of deceivers. As further hypothesized, the strategically relevant criteria were found to carry strategic meaning of either positive or negative valence.

Regarding cognitive criteria, the results showed that participants tended to ascribe strategic meaning of different valence to them. This finding, in fact, supports our predictions since at closer inspection a clear difference between the two dimensions of cognitive criteria was indeed visible: Taking only statistically significant value ratings into account, all ratings for memory-related (group 1) criteria were of positive valence. Among these criteria the effect sizes were of medium strength (*d* > 0.5; Cohen, 1988), except for *temporal information* (*d* = 0.19). The value ratings for 6 of the 10 memory-related criteria were statistically non-significant, among which the strategic ratings of 3 criteria (*own thoughts, sensory impressions, attribution of perpetrator's mental state)* were tentatively negative. Put another way, if participants attributed strategic meaning to a criterion from group 1 (in terms of value ratings that differed significantly from “0”), the valence was exclusively positive. In sharp contrast, for script-deviant (group 2) criteria all ratings assumed significant negative values, with the strength of their effect sizes ranging from small (*d* > 0.2; Cohen, 1988) to large (*d* > 0.8; Cohen, 1988).

Regarding motivational criteria (strategy-based criteria; group 3), the value ratings of all criteria differed significantly from “0.” For 7 of the 9 criteria, these ratings were negative—with predominantly large effect sizes—and hence in line with our predictions. Other than predicted and deviating from the forensic assumptions about the strategic meaning of motivational criteria, *self-deprecation* {*M* = 0.36, *SD* = 1.18, *t*_(134)_ = 3.57, *d* = 0.31, 95% CI [0.16, 0.56], *p* < 0.001} and *pardoning the perpetrator* {*M* = 0.44, *SD* = 1.05, *t*_(134)_ = 4.93, *d* = 0.42, 95% CI [0.27, 0.62] *p* < 0.001} obtained significant positive values, suggesting that participants intended to integrate rather than avoid these contents.

## Discussion

Our findings indicate that on the motivational level, most CBCA criteria are clearly distinguishable in criteria that liars are inclined to integrate vs. criteria that liars are inclined to avoid in their statements. For half of the 18 strategically relevant criteria (as defined by value ratings differing significantly from “0”) we obtained effect sizes of at least medium strength, which may be interpreted as evidence for the practical significance of these findings. Furthermore, even though the three-group structure of the revised model goes beyond simply dichotomizing the criteria in bearing positive vs. negative valence, it nonetheless yielded a widely homogenous pattern of strategic value ratings. In comparison to the 5-group classification used in the two previous studies (Niehaus et al., 2005; Niehaus, 2008), a considerably higher degree of compatibility within each criteria group was observable. For illustration purposes, Table 5 applies the results of all three studies to the structure of the revised model, enabling viewers to graphically examine to what extent the studies' results tally with each other.

**Table 5:**
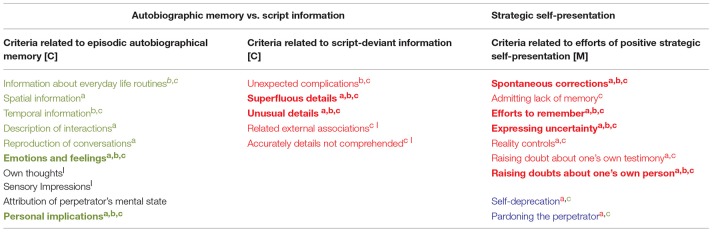
Strategic value ratings as obtained from all three studies^d^.

*[C], Cognitive criteria; [M], Motivational criteria*.

I *Investigated only in the current study*.

p < 0.05:

a study of Niehaus et al. (2005)

b study of Niehaus (2008)

c *current study*.

*Criteria in green font color received significant positive ratings in at least one of the three studies (and no significant negative ratings in any of the other studies). Vice versa, criteria in red font color received significant negative ratings (and no significant positive ratings in any of the other studies). If a criterion received both significant positive and negative value ratings across different studies, a blue font color was applied. Additional bold font highlights that the criterion was rated significantly across all three studies*.

d *For reasons of clairty, the structure presented in this article differs slightly from the version originally presented by Volbert and Steller (2014); see footnote 5 for details. Also, Table 5 excludes criteria that were previously investigated by Niehaus et al. (2005) and Niehaus (2008), but that the authors of the revised model had allocated to the “statement as whole” category (quantity of details, unstructured production) or not adopted at all (justifying memory gaps/uncertainties, spontaneous clarifications)*.

Inspecting the valence of the strategic ratings on group level, the degree of compatibility appears to be lowest for memory-related criteria of group 1. That is, in our study the strategic ratings of more than half of the criteria in this group failed to reach significance and in part showed a directional tendency opposite to the direction of the strategically relevant criteria. If collectively examined however, *attribution of perpetrator's mental state* remains the only criterion to which no (positive) strategic meaning was attributed in any of the three studies^4^ (see Table 5). Nonetheless, the overall picture for criteria of group 1 remains less consistent than for the criteria of the other two groups, as positive ratings that were significant in *each* of the three studies (2 out of 7 criteria) were the exception rather than the rule.

Viewed the other way around however, it is crucial to note that none of the three investigations yielded significantly negative ratings for memory-related criteria, a pattern which sharply contrasts with the results found for script-deviant and strategy-based criteria. Motivational considerations are therefore of little avail in ascribing diagnostic value to memory-related criteria, as the underlying rationale requires that liars would typically avoid such contents. Instead, only considerations on the cognitive level may explain why these specific criteria are more likely to occur in true than in fabricated statements, implying that when lying certain contents are more difficult to produce than when telling the truth.

Regarding script-deviant criteria of group 2, our results showed that they consistently carry negative strategic meaning for laypersons. These results correspond well with the findings previously reported by Niehaus et al. (2005) and Niehaus (2008), as illustrated by Table 5 (significant negative ratings across all three studies^5^ for 2 out of 3 criteria). In contrast to memory-related criteria, motivational considerations thus appear relevant when assessing the diagnostic value of script-deviant criteria, considering that deceivers typically intend to avoid their production.

As pointed out before, both memory-related (group 1) and script-deviant (group 2) criteria were originally classified as being cognitively-related. Interestingly, even though the newly-made distinction of memory-related vs. script-deviant criteria was originally deduced from considerations on the cognitive level—assuming that different cognitive processes underlie their production—, the hypothesized differences seem to translate to the motivational level as well. In this way, the obtained pattern of strategic value ratings clearly corroborated the utility of distinguishing between two groups of cognitive criteria.

Other than group 1 and group 2 criteria, strategy-based (group 3) criteria are classified as motivational criteria, which implies that their validity depends largely on the assumption that deceivers out of strategic reasons avoid producing them. While our results indeed showed consistent negative ratings for 7 of the 9 criteria (with 4 of them being rated significantly negative across all three studies; see Table 5), participants in our study attributed positive strategic meaning to the criteria *self-deprecation* and *pardoning the perpetrator*. Participants in the investigation of Niehaus et al. (2005) on the other hand had rated the same criteria significantly negative. Possibly, the discrepant findings between the two investigations might be attributable to their variations in context: While the story outline of the current study revolved around a rather ordinary every-day work situation, the context in Niehaus et al.'s (2005) study bore graver ramifications and entailed false allegations of sexual rape. Such relationships between context and valence of the rating would, in fact, correspond well with the proposition of Niehaus et al. (2005), predicting that the negative strategic meaning of *self-deprecation* and *pardoning the perpetrator* is dependent on sufficient contextual gravity to render elements related to self-criticism unconceivable. Within less severe contexts on the other hand (i.e., scenarios typically used in laboratory studies, such as accusations of minor theft or insurance fraud) laypersons would ascribe positive strategic value to these elements, believing to make them appear more amiable and trustworthy. It is not clear however whether the negative strategic ratings reported by Niehaus et al. (2005) primarily reflect the sexual connotation of the context or rather the severe ramifications associated with it, leaving open the question under which specific circumstances the criteria could be valid indicators for truthfulness. Furthermore, empirical findings from several laboratory studies appear to dispute their validity in indicating true testimony. Amado et al. (2016) for instance identified in their meta-analysis *self-deprecation* and *pardoning the perpetrator* as the only criteria that failed to discriminate between true and fabricated statements, while Vrij (2008) even found that in two out of ten studies, *self-deprecation* appeared significantly more often in fabricated than in true statements. Crucially though, the design and nature of laboratory studies typically vary in important aspects from forensic interrogation settings (Volbert and Steller, 2014), including but not limited to the gravity of the context in which the interview takes place (Burgoon, 2015). Inferring from these findings that *self-deprecation* and *pardoning the perpetrator* are by or in themselves unsuitable in indicating true testimony may therefore be premature. Instead, further investigation seems warranted to explicate the exact contingencies under which laypersons are inclined to avoid rather than promote their production.

At least to a weaker degree, if viewed collectively the studies' results may hint at additional criteria that in their strategic meaning are context-dependent. Concerning memory-related (group 1) criteria for instance, context-dependency may explain why across studies participants rated the first five criteria uniformly within the two studies that introduced a nearly identical story outline, but differently so in the investigation that referred to a scenario with considerably graver ramifications (Niehaus et al., 2005). From a purely theoretical perspective, it seems further possible that the strategic meaning of strategy-based (group 3) criteria may depend on the underlying context as well. For instance, some of these criteria pertain to contents that in true accounts reflect the expression of genuine mnemonic processes, such as *admitting lack of memory* or *efforts to remember*. From their own experience liars may be well aware that memories fade with time, and thus may ascribe rather positive strategic meaning to these contents when the event in question dates back enough years in time. Since all three studies introduced scenarios in which only brief time periods lay between statement and the event to be imagined, their paradigms would be unsuitable for detecting such forms of context-dependency. Future studies could examine this issue by implementing scenarios that differ in regards to the length of time that had passed between event and statement deliverance.

### Limitations

Some important limitations of our study deserve attention. As we made use of a questionnaire, we cannot be sure whether participants correctly understood every example that we provided for illustrating the criteria. Furthermore, our conclusions about content-related deception strategies are based on averaged findings that may not apply equally well across individual cases. For instance, Niehaus (2008) found that the specific content-related deception strategies vary between different age groups, suggesting that the developmental stage of a person may mediate the strategic meaning he or she ascribes to a criterion^6^. Most importantly, we only investigated how lay people rate the strategic meaning of the criteria in theory but did not test in which ways their content-related deception strategies translate to the practical level. Considering that the potential outcome for the liar can highly affect his or her behavior associated with deception (Porter and ten Brinke, 2010), it seems reasonable to assume that participants' hypothetical use of content criteria in fictitious scenarios may differ from their actual verbal performance in real-life forensic settings. Future research would first need to explore or even manipulate the deception strategies of participants (i.e., pointing out to them the strategic meaning of criteria from the perspective of forensic practitioners), and subsequently motivate participants to successfully deceive within an ecologically valid, high stakes interrogation setting^7^. Such an approach would allow examining the relationship between the strategic meaning that statement providers ascribe to a criterion and the criterion's subsequent occurrence in their fabricated statements.

### Conclusions

The current study demonstrated that CBCA criteria differ in their strategic meaning and that the three-dimensional structure of the revised model of Volbert and Steller (2014) is suitable for representing these differences. Few exceptions (such as *self-deprecation* and *pardoning the perpetrator* carrying strategic value opposite to the valence of their respective group) notwithstanding, our group-based predictions regarding the strategic meaning of the criteria were largely confirmed. That is, laypersons tended to rather ascribe positive strategic meaning to criteria related to episodic autobiographical memory (group 1) but tended to ascribe negative strategic meaning to criteria related to script-deviant information (group 2) and efforts of strategic self-presentation (group 3).

In practical terms, our results then provide valuable input for forensic practitioners in appraising the diagnostic value of the criteria: The fact that deceivers typically intend to refrain from simulating script-deviant (group 2) or strategy-based (group 3) criteria strengthens their validity in indicating true statements. In contrast, no such avoidance inclinations are to be expected for memory-related (group 1) criteria, implying that the mere presence of these criteria does not automatically support a statement's truthfulness. Such generalized guidelines can only be of heuristic value however, since positive strategic meaning is rather a prerequisite than actual indication for a criterion's emergence– whether the criterion occurs in the fabricated statement then depends on the statement giver's ability to produce such content (primary vs. secondary deception; Köhnken, 1990). More elaborate assessments of a criterion's diagnostic value thus necessitate additional insight about the cognitive component; above all, knowledge about the cognitive difficulty associated with the criterion's production is required. Such insight combined with our established knowledge about the strategic meaning of the criteria would then constitute a solid foundation for optimally assessing their diagnostic value.

## Data availability

The raw data supporting the conclusions of this manuscript will be made available by the authors, without undue reservation, to any qualified researcher.

## Ethics statement

This study was carried out in accordance with the Ethical Guidelines of the German Psychological Society (DGPs). All subjects gave informed consent in accordance with these guidelines. Ethical approval was not deemed necessary as there was no foreseeable risk of harm or discomfort for participants.

## Author contributions

RV, SN, and SW: contributed to conception and design of the study; SW: carried out the experiment and organized data acquisition; BM and SW: performed the statistical analysis; BM: wrote the first draft of the manuscript; BM and RV: wrote sections of the manuscript; RV, BM, and SN: contributed to manuscript revision. All authors read and approved the submitted version.

### Conflict of interest statement

The authors declare that the research was conducted in the absence of any commercial or financial relationships that could be construed as a potential conflict of interest.

## Footnotes

^1^In total, lying participants reported five categories of strategies regarding the verbal content of their statements, with “providing a detailed story” and “avoid saying things that were not true” being the categories most frequently mentioned (each being mentioned by 22% of the relevant sample). As pointed out by Hartwig et al. (2007), these findings were limited to qualitative analyses and thus are best to be interpreted as a starting point for future research.

^2^The study was conducted in German language.

^3^See Appendix 1 for a display of the entire story as presented to participants.

^4^The criteria *own thoughts* and *sensory impressions* were only investigated in the present study, so no comparisons to the other two studies can be made.

^5^The criteria *related external associations* and *accurately reported details misunderstood* were only investigated in the present study, so no comparisons to the other two studies can be made.

^6^For explanatory purposes, we tested whether the strategic value ratings in our study differed between students (*n* = 66) and working professionals (*n* = 55). While the difference in age between students (*M*_age_ = 23.70, *SD* = 3.70) and working professionals (*M*_age_ = 35.00, *SD* = 11.98) was significant, *M* = −11.30, *t*_(62.60)_ = −6.73, *p* < 0.001, none of the one-way ANOVAS conducted for each criterion yielded a significant difference in the value ratings between groups. Presumably, age differences in the assignment of strategic value to certain CBCA criteria are linked to different developmental stages (i.e., adults versus children; see Niehaus et al., 2005). Despite the difference in age, both groups in our study consisted of adults, rendering the existence of different developmental stages unlikely.

^7^For definitions of high stakes, experimentally realistic lie detection scenarios as well as their effects on lie detection accuracy see O'Sullivan et al. (2009).

## Appendix 1: display of the entire story as presented to participants (translated from german)

*Dear participants*,

we are interested in your strategies of how to make a fabricated story appear credible. There are no “wrong” answers; we will not assess your answers on moral grounds, nor do we want to know if you would pursue such strategies in real-life. Please read the instructions carefully and please answer all questions in their order of appearance. For your responses, please put yourself into the perspective outlined in the following story:

For 6 weeks you have been holding a job position you had desired before for a long time. However, within these first few weeks some things went wrong: Twice already you have shown up to work late. Your boss is quite irritated about this, and since you are still on probation you should by no means show up late a third time. Your boss made clear that if this should happen again, you will lose your position. Yet, it happens again: You are oversleeping in the morning! You hurry to the tram, where you happen to meet your neighbor Mr Schneider, who is quite an irritable fellow. Once he had even punctured the tires of your bike. Running into this guy was the last thing you needed now!

During the tram ride you are contemplating how you can explain yourself best to your boss, and looking at your maliciously smirking neighbor you come up with the following story:

“*This morning, right before I was about to leave for work, I went down to the cellar to fetch a folder with work-related documents. They contain my personal notes that I intended to use for work. So, here is what happened: I was unlocking the door to the cellar, and as always, I left the key in the lock. Upon entering the cellar, I recognized some noise behind me. As I turned around, Mr. Schneider - my neighbor, who constantly causes trouble - was standing outside. He quickly moved forward and locked up the door to the cellar. I had been calling and pounding on the door for quite a while until finally an unfamiliar person showed up and unlocked the door. Luckily, the key was still left on the outside lock. The unfamiliar person moved on, and I quickly made my way to work.”*

*After having told this story to your boss, your boss remarks: “Oh, Mr Schneider, I do know this guy! He works as doorman for the company of my wife! Maybe I should talk to him, what he just did is hardly acceptable.” Then, your boss instructs you to tell him again what had happened, as precisely as possible. He lets you know that he will not talk to Mr Schneider if he finds your story set out convincingly. Otherwise however, in case he will have doubts about your story, he will talk to Mr Schneider for clarification. Clearly, it is crucial now that your boss believes your story. You certainly do not want to lose your job! You even had wrongly accused another person- Mr Schneider would, of course, deny the allegations, so your only hope is to convince your boss with your story. Otherwise, you will lose your job for sure*.
